# Titanium-Based Metasurfaces for Optoelectronics

**DOI:** 10.3390/nano14010056

**Published:** 2023-12-25

**Authors:** Stella Kavokina, Vlad Samyshkin, Junhui Cao, Andrey Abramov, Anton Osipov, Samuel Pier Essaka, Nazrullo Khalimov, Dmitry Bodunov, Alexey Kavokin

**Affiliations:** 1 School of Science, Westlake University, 18 Shilongshan Road, Hangzhou 310024, China; caojunhui@westlake.edu.cn (J.C.); a.kavokin@westlale.edu.cn (A.K.); 2 Institute of Natural Sciences, WIAS 18 Shilongshan Road, Hangzhou 310024, China; 3 1D-Laboratory, Vladimir State University Named after AG and NG Stoletovs, Gor’kogo Street 87, Vladimir 600000, Russia; simplevladius@mail.ru (V.S.); abramov.andrey.1997@gmail.com (A.A.); osipov@vlsu.ru (A.O.); nazutojake99@gmail.com (S.P.E.); nazrullonazar2000@gmail.com (N.K.); bodunov-2002@mail.ru (D.B.)

**Keywords:** titanium dioxide, laser action, gold nanoparticles, sp-carbon, metasurface, photodiode

## Abstract

We report on the fabrication method that enables the development of transparent conductive metasurfaces capable of the resonant absorption of light in specific frequency bands. The approach is based on embedding linear sp-carbon chains and metallic nanoparticles in a porous matrix of titanium dioxide (TiO_2_). We develop a blading technique for the formation of a periodical grating of TiO_2_ microtubes at the macroscale. The method allowed us to maintain the periodicity of an array of microtubes with an accuracy of ±5%. Tuning the diameter of the tubes and the concentration of metallic nanoparticles, we achieved the regime of strong resonant absorption of the fabricated complex metasurface in the visible range. Computer simulations helped revealthe regime of TE/TM-polarized laser pumping that allowed for the most efficient transformation of light energy into electric current flow. In the studied structures, the sp-carbon clusters embedded inside transparent titanium dioxide tubes play the role of atomic wires. The interplay between efficient conductivity through carbon wires and the plasmon-enhanced absorption of light allows the design of photodiode structures based on periodical metasurfaces and characterized by highly selective optical sensitivity.

## 1. Introduction

Modern optoelectronics is developing at a high pace mostly due to the invention of new materials that enable the efficient engineering of optical and electronic properties of device structures [1]. The engineering of optical properties is achieved by the use of metamaterials. In metamaterials, both the real and imaginary part of the refractive index may be tuned by combining metallic and insulating elements arranged in a sort of supercrystal lattice [2,3,4]. The engineering of electronic properties is being realized via the fabrication of multilayer structures composed of various two-dimensional crystals, such as graphene, transition metal dichalcogenides, h-BN, black phosphorus, etc. [5,6,7]. Still, the challenge remains of combining desired electronic and optical properties within the same thin crystal layer. For device applications, such a layer must be deposited on a substrate, put in contact with electrodes and protected by a transparent cover layer. Metamaterials rarely conduct an electric current, while some exceptions associated with the use of carbon nanotubes are indeed possible [8]. Also, conductors are rarely optically transparent. In order to circumvent this apparent obstacle, one needs to create a conductive but transparent metasurface. On the top of this, ideally, this metasurface should be able to efficiently absorb light of a specific wavelength and efficiently convert it into electric current. Such a “dream metasurface” would enable the fabrication of a new generation of photo-diodes and photovoltaic elements. This study presents the fabrication technique that enables the creation of a conductive transparent metasurface. The metasurface is based on an array of titanium dioxide microtubes. Gold nanoparticles and monoatomic carbon wires are embedded in microtubes, making them conductive and altering their optical properties [9]. We characterize the fabricated structures using optical spectroscopy methods and propose the design of a photo-diode based on the newly fabricated metasurface.

The fabrication of such a “dream” metasurface requires a three-stage procedure. First, we synthesize a matrix of porous TiO_2_; second, we dope it with conductive sp-carbon chains and gold nanoparticles; third, we roll it into a periodic array of microtubes.

Titanium dioxide (TiO_2_) is a key element of the metasurface under study. TiO_2_ and its composites are promising for photocatalytic and photoelectrochemical applications that imply the conversion of solar energy into electricity [10]. TiO_2_ is characterized by a large energy gap that is about 3.0 and 3.2 eV for rutile and anatase forms, respectively. This makes TiO_2_ transparent in the visible light frequency range, which prevents the absorption of visible light and the efficient conversion of solar energy [11]. TiO_2_ only shows significant optical activity in the UV range characterized by wavelengths less than 360 nm [12]. This is why UV sources are required for the pumping of TiO_2_ crystals in opto-electronic devices. This represents a significant limitation to the application of TiO_2_ in optoelectronics. An alternative to the use of UV sources is the optimization of the optical properties of TiO_2_-based structures via the implantation of metal ions/atoms, non-metallic compositions, or dyes [10,11,12,13]. Still, in order to be suitable for the realization of a transparent solar device, several requirements need to be met. In particular, in the device structure, not only the active layer but also all intermediate elements must be transparent. The coexistence of high photocatalytic efficiency with sufficient optical transmittivity in the structure is difficult to achieve. Indeed, the higher the transparency of the device, the lower percentage of the solar energy absorbed in its active layer and the lower energy outcome generated by the device. Here, we offer a method that allows us to bypass this formidable challenge. We design a periodic metasurface that enables one to combine the efficiency of resonant plasmon absorption provided by gold nanoparticles and the efficient conductivity of sp-carbon wires incorporated inside the TiO_2_ porous matrix. Based on this achievement, we also propose a concept of a photo-diode based on periodically oriented arrays of TiO_2_ tubes.

The manuscript is organized as follows. Section 2 describes the materials used to fabricate the designed metasurfaces and presents the fabrication method in detail. Section 3 presents the scanning electron microscopy (SEM) images of the fabricated structures as well as their optical absorption spectra. This section also contains the results of the numerical modeling of the optical spectra and light field distribution in the structure. Section 4 presents the discussion of the proposed device concept. Section 5 summarizes the results of our study. In the Appendix A, we present the supplementary SEM image of TiO_2_ films taken prior to the blading and fabrication of the array of tubes and the TEM image of the gold and carbon nanostructure admixture.

## 2. Materials and Methods

**Figure A1:**
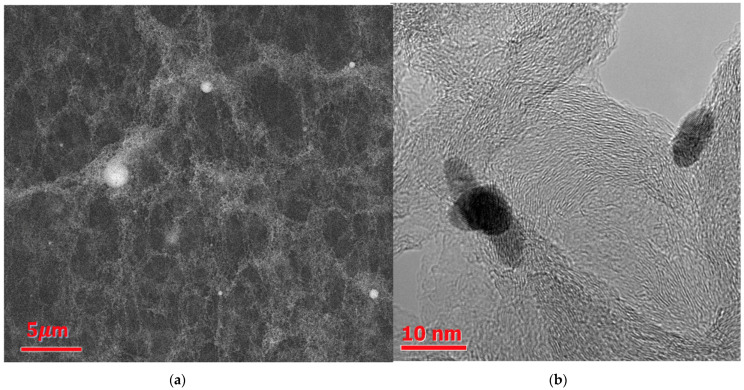
(**a**) SEM image of the initial porous titanium dioxide film; (**b**) TEM image of the free-space film of sp-carbon chains stabilized with gold nanoparticles. The water solution of carbon chains was sedimented as a top layer on the porous titanium dioxide film shown in (**a**) to improve the optical and electronic properties of the multilayer structure. Due to the high porosity of titanium dioxide, carbon chains and gold NPs diffuse inside the TiO_2_ matrix, thus forming a hybrid material that can be used for the formation of a metasurface. Once this is carried out, the resulting structure is ready to be wrapped in order to form an array of rolls, as Figure 1 shows.

We used pure titanium bulk crystals produced by LTd Ligamet (Moscow, Russia) as a target for laser ablation [14,15]. A titanium crystal was irradiated by nanosecond laser pulses from IR-source L-Designer made by Ateco (Moscow region, Russia). The laser pulses were of a wavelength of 1064 nm with a pulse duration of 60 ns and with a frequency of 20 kHz, and they were used to illuminate the Ti bulk precursor for 240 s in order to fabricate a 4 μm thick film of TiO_2_ on a substrate (see Figure A1a). We employed polished quartz glass as a substrate for the deposition of the synthesized titanium dioxide film. To enhance metal oxidation during the laser treatment, we either blew in extra air/oxygen or applied an external magnetic field. Both methods led to the active interaction of plasma erosion torch vapors with the gas media. As a result, the porous thin film of TiO_2_ was created, as shown in Figure A1a. It was used for further processing and optical studies.

In the second stage of the fabrication procedure, we used the sputtering method in order to engineer the optical and electronic properties of the porous TiO_2_ matrix. The distilled water solution of sp-carbon chains stabilized with gold nanoparticles with a concentration of about 5% (see Figure A1b) was sedimented in a layer on top of TiO_2_. Due to the high porosity of TiO_2_, gold and carbon nanostructures then diffused into the TiO_2_ layer, forming a hybrid structure containing nanoscale metallic and carbon elements embedded in the host structure of TiO_2_. It is important to note that due to the presence of sp-carbon nanowires, the resulting metamaterial became conductive. The presence of gold NPs strongly affected its optical properties, as we discuss below.

The colloidal solution that was used to enrich the TiO_2_ matrix with gold and carbon was prepared in accordance with the method described in [16,17]. We refer to the resent publications [16,18,19,20] for details on the electronic and optical properties of linear sp-carbon chains. For our study, it is important to note that linear carbon allotropes do not absorb light in the visible range. This is why, in the literature, they are frequently referred to as white carbon. On the other hand, gold nanoparticles feature pronounced resonances in the optical absorption spectra that are associated with plasmon absorption. These plasmonic features manifest in the light-pink staining of the solution, which can be seen by the naked eye.

**Figure 1:**
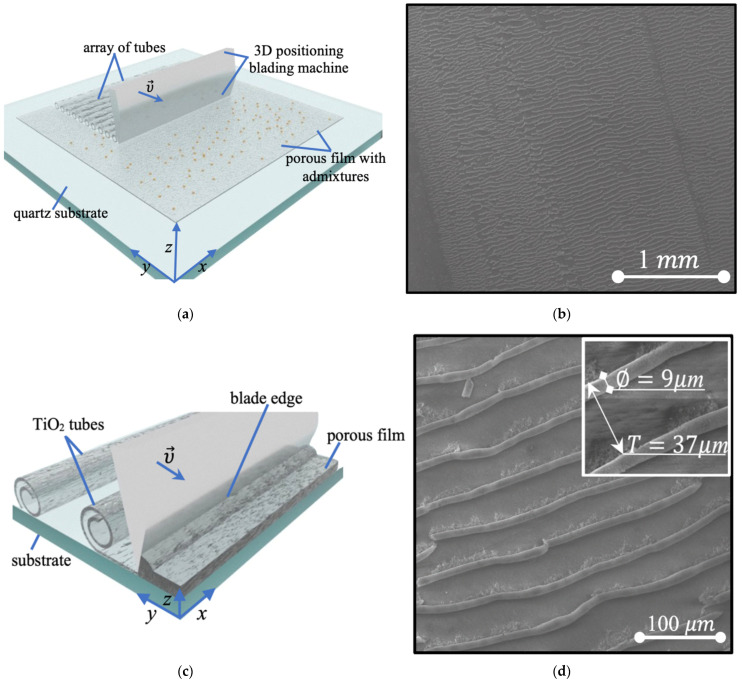
The blading method employed for the realization of a periodical array of TiO2 tubes. (**a**) A schematic illustrating the general concept on a polished quartz substrate with a layer of porous TiO_2_ and a sprayed admixture, particularly sp-carbon chains stabilized by Au NPs; the blade acts mechanically, moving at a constant height in the positioning stage. The tubes are wrapped after contact with the blade edge. (**b**) The resulting SEM image of the rolled surface after blading on a millimeter scale. (**c**) A detailed illustration of the area of contact between the deep blade and porous TiO2 film. (**d**) An SEM image of the fabricated array of TiO_2_ tubes on a micron scale. The geometrical parameters most suitable for the photodevice applications are indicated in the image.

In the third and final stage of the fabrication procedure, the layered film was mechanically wrapped into an array of microtubes by means of micro-blade machining. The blading technique employed for the formation of an array of TiO2 tubes is illustrated in Figure 1a,c. The tip of a blade located on the 3D micro-positioning stage was held parallel to the substrate surface. The resulting diameter of rolled tubes and the period of fabricated grating were determined by moving stage parameters. Its motion in light contact with a surface along the *y* or *x* axis resulted in the deformation of the thin film. Once an edge of the blade was buried at least 1 μm deep into the volume of the film, blading gave rise to the film folding into multilayer tubes (see Figure 1c). The average diameter of rolls periodically located and oriented perpendicular to the blade strongly depended on the blade penetration depth and on the step of the 3D positioning stage. The processed rolls after blade passing were subjected to structural changes. In Figure 1c, one can see the resulting few mm scale area covered with rolled tubes, oriented along the blade edge. The transition zone corresponding to the area of contact between the initial thin film and the blade edge is seen on the left-hand side of Figure 1c. In every stage of the fabrication procedure, we controlled the structure under study by means of scanning electronic microscopy (SEM) and optical spectroscopy. The optical transmission spectra of the fabricated structures were detected via the use of the scanning spectrophotometer Unico 2804 by United Products & Instruments Incorporated (Dayton, FL, USA) and the spectrophotometer PerkinElmer Lambda 1050 (Shelton, CT, USA).

The size of gold NPs was controlled using the dynamic laser scattering device Horiba SZ100 (Kyoto, Japane). For the detailed study of the geometry of produced periodic arrays, we performed SEM imaging using Quanta 200 3D produced by FEI (Hillsboro, OR, USA). Optoelectronic transformation efficiency was measured with a Fluke multimeter (Everett, WA, USA) in the regime of photostimulation at a wavelength of about 500–520 nm, which corresponds to the plasmon resonances in gold nanoparticles of different sizes [21]. We used second harmonic generation at a central wavelength of 532 nm for the L Designer laser system (Moscow region, Russia). Thorlabs (Newton, NJ, USA) optical components were employed as light filters and polarizers to form a pump beam in the photo stimulation study. For the detailed study of the morphology of sp-carbon chains and gold NPs, we performed high-resolution transmission electron microscopy using FEI Titan3 with a spatial resolution of up to 2 $\dot{A}$.

## 3. Results

### 3.1. Formation of a Periodical Array of TiO_2_ Tubes

SEM images of the fabricated structures are shown in Figure 1b,d. Figure 1b shows the view of an array of TiO_2_ microtubes after blading on a millimeter scale. One can see that the method allowed us to maintain the periodicity of an array of microtubes with an accuracy of ±5%. We note that the observed inhomogeneities and the distribution of the period of the array of TiO_2_ tubes on a millimeter scale are associated with the imperfection of the blade, which may be technically amended further. Figure 1d presents a detailed view of a fragment of the grating. This image allows us to extract the geometrical parameters of the grating. We note that this specific choice of parameters provides the most efficient light-to-electric current conversion rate. Specifically, the period of the grating is 37 µm, while the diameter of each tube is about 9 µm.

### 3.2. The Optical Spectroscopy Study

**Figure 2:**
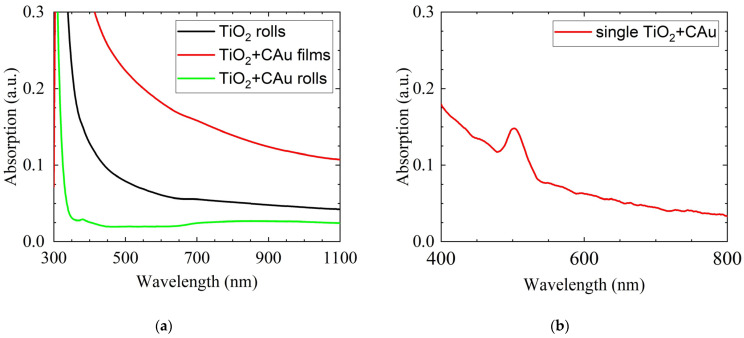
Optical absorption spectra. (**a**) The integral optical absorption intensity collected from the sample area of a few square millimeters. The red curve corresponds to the absorption of an initially fabricated thin film of TiO_2_ + CAu, the black line shows the absorption of an array of pure TiO_2_ tubes, and the green line shows the absorption of an array of TiO_2_ + CAu tubes. (**b**) The absorption spectrum taken from a single tube of TiO_2_ + CAu.

We studied the optical spectra of the initially fabricated TiO_2_ film of a 4 μm thickness with carbon and gold admixtures (see the red line in Figure 2a), and then repeated the study after having accomplished the blading structurization of the surface. This procedure enabled us to visualize the absorption spectra modification caused by the formation of an array of TiO_2_ tubes (see the green curve in Figure 2a). The black curve in Figure 2a is the absorption spectrum of an array of pure TiO_2_ tubes shown for comparison. One can see that there is a pronounced reduction in absorption (or enlightenment) in the arrays of tubes as compared that in case of the thin film. First of all, this is due to the formation of spatial gaps between tubes, which switches off absorption in significant areas of the sample. Second, the admixture of carbon and gold affects total absorption but does not lead to the appearance of resonant features in spatially integrated spectra containing less than 5% of carbon and gold admixtures. In contrast, if the signal is collected from a single tube, the plasmon absorption features can be clearly seen (Figure 2b).

### 3.3. Simulations of the Absorption Spectra

**Figure 3:**
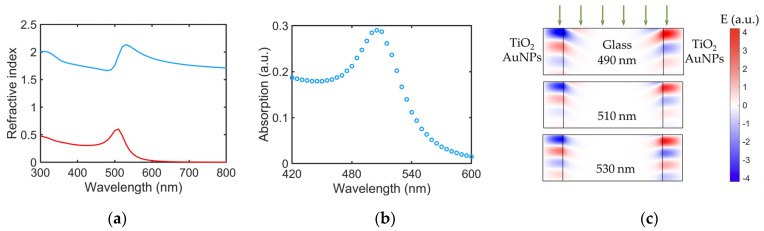
(**a**) The effective refractive index of the TiO_2_ + Au NP hybrid material calculated from the Maxwell–Garnett equation, where the blue and red lines indicate the real and imaginary refractive index, respectively. (**b**) The absorption spectrum calculated for the structure shown in Figure 1d. The peak located at 507 nm can be attributed to the plasmon resonance of Au NPs, where the imaginary part of the refractive index of TiO_2_ + Au NP stripes also reaches its maximum. (**c**) The real space distribution of the electric field of light incident on the structure at different wavelengths. The green arrows indicate the direction of the incidence of light.

We simulated the optical spectra of a periodical structure made of TiO_2_ tubes doped with carbon wires and Au NPs by solving the wave equation $\nabla \times \nabla \times E=k^{2}E$ using the finite difference method. We assumed a normal incidence with the polarization of light perpendicular to TiO_2_ tubes. The effective refractive index (Figure 3a) of the TiO_2_ tube/Au NP structure is estimated with the Maxwell–Garnett equation, $\frac{\epsilon -\epsilon_{TiO_{2}}}{\epsilon +\epsilon_{TiO_{2}}}=P\frac{\epsilon_{Au}-\epsilon_{TiO_{2}}}{\epsilon_{Au}+\epsilon_{TiO_{2}}}$, where ϵ is the dielectric constant of the hybrid material, TiO_2_ + Au NPs, $\epsilon_{TiO_{2}}$ and $\epsilon_{Au}$ are the dielectric constants of TiO_2_ and Au, respectively, and P is the percentage of gold. The spectral dependence of the refractive indices for gold was retrieved from ref. [22]. This analysis confirms that, in Figure 3b, starting from the spectral range of 400–500 nm, the decrease in background absorption is due to the decrease in the imaginary part of the refractive index of the hybrid material. At a wavelength of around 507 nm, absorption suddenly increases but then it decreases again, in good agreement with the experiment (Figure 2b). This absorption peak is associated with the plasmonic resonance of the Au NPs, which coincides with the maximum of the imaginary refractive index shown in Figure 3a. The distribution of the electric field near the resonance frequency is shown in Figure 3c. Due to the high refractive index of the hybrid material in comparison to that of the glass substrate, the maxima of the electric field intensity are located in the TiO_2_ + AuNPs stripes.

## 4. Discussion

**Figure 4:**
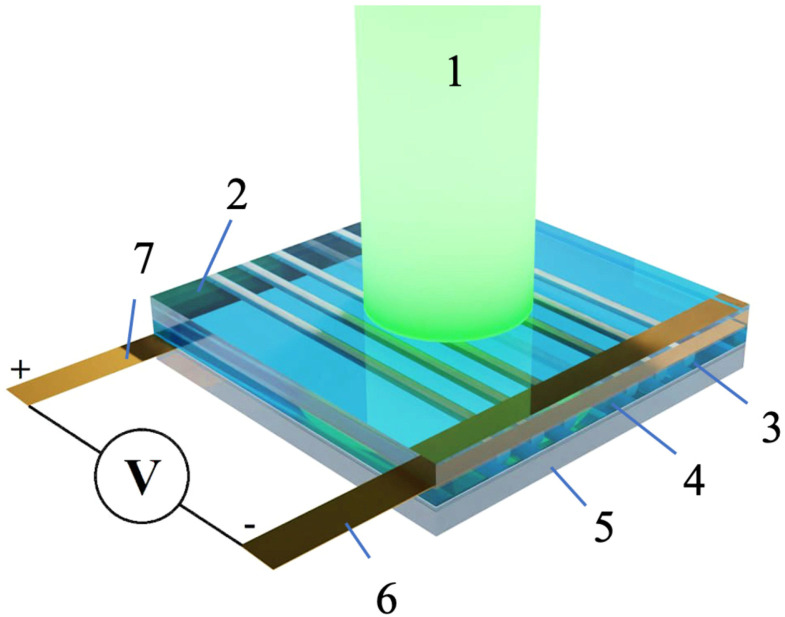
The proposed scheme of a photo-diode based on an array of TiO_2_ tubes doped with carbon wires and gold nanoparticles. The key components of the device are indicated by numbers in the drawing. Briefly, 1 stands for the pumping laser beam, 2 is the quartz glass substrate, 3 is the cuvette filled with the electrolyte, 4 shows the metasurface formed by an array of porous TiO_2_ microtubes with embedded carbon nanowires and gold nanoparticles, 5 shows the ITO glass substrate, and 6 and 7 are the top and bottom electrodes, respectively.

The results of our calculation clearly show that an array of TiO_2_ microtubes doped with sp-carbon chains and Au nanoparticles can be considered a quasi-transparent medium. Such a medium is expected to be highly promising for light–electron energy conversion. As follows from the simulations above, the optical pumping of such structures must obey certain conditions. Namely, the pumping light should be polarized normally to the tubes of TiO_2,_ and its wavelength must be close to that of plasmon resonance in gold nanoparticles (507 nm) in order to maximize absorption. It is important to note that Au nanoparticles, incorporated in a porous TiO_2_ matrix, act as donors of electrons in the case of the resonant optical excitation of plasmon modes hosted by these nanoparticles. A conducting network of sp-carbon wires with single-electron bonds with Au NPs assures the high electric conductivity of each tube. The conductivity in this hybrid metal–organic system may be induced by laser light, as has been demonstrated recently [23,24,25,26]. The origin of this effect is the electronic transfer between gold NPs and carbon nanowires triggered by the excitation of plasmon modes in gold NPs. In order to capitalize on the advantages offered by the unique optical and electronic properties of TiO_2_ tubes embedded with gold and carbon wires, we propose a concept of a photo-diode based on an array of such tubes, as Figure 4 shows.

The proposed device structure is composed of a top cover layer, an active layer and a bottom layer. The top layer consists of an optically transparent quartz glass substrate that is essential for optimizing optical pumping conditions. The bottom layer is made of indium tin oxide glass substrate, which provides conduction and is transparent for the visible light base that is essential for the working environment of the device. The periodic array of TiO_2_ tubes doped with gold NPs and sp-carbon chains constitutes an active layer clamped between the substrates. The substrates are confined by L-shaped electrodes placed along the perimeter of the device. The electrodes together with the conducting layers form a closed electrical circuit composed of the vertical and horizontal parts. The bulky electrodes contain cavities filled with a strong transparent electrolyte. The junction between the array TiO_2_ tubes containing the incorporated gold and carbon admixture (an n-doped layer) and ITO glass (a p-doped layer) represents a p–n junction. The area illuminated by the laser light in Figure 4 represents an optical radiation receiver that converts the energy of incident photons into an electric current flowing from the top to the bottom of the structure through the p–n junction.

We undertook the first study of a device prototype characterized by an active area of 4 × 4 cm^2^ and an irradiation power of about 0.06 W/cm^2^. We observed the light-induced drop in the electrical resistance of the device from 500 Ohm to 270 Ohm. Further studies are under way to extract the spectral dependence of the light-induced conductivity of the device, and find the transition time constant and other parameters of the dynamic processes involved. These preliminary studies show that the proposed device concept can be practically implemented and it can also be generalized to enable excitation frequencies within the visible spectrum. Various modifications of the proposed design would meet the needs of a variety of applications in photonics.

## 5. Conclusions

We developed an experimental method that enables the fabrication of a transparent conductive metamaterial capable of efficiently absorbing light in a specific narrow frequency range. The combination of optical transparency, electronic conductivity and resonant absorption of light was achieved through a two-stage process consisting of, first, the surface deposition of a thin film made of titanium dioxide with embedded sp-carbon chains and gold nanoparticles, and, second, the mechanical blading of the deposited film, leading to the formation of an array of microtubes. The optical spectroscopy of the fabricated structure revealed a strong resonant feature at the plasmon frequency of gold nanoparticles. We anticipate efficient energy transfer between the incident light and the ensemble of charge carriers at this frequency. The effect may be used in photo-diodes, the active layer of which would consist of a metasurface formed by an array of titanium dioxide microtubes sculptured with monoatomic carbon chains and gold nanoparticles. We propose a specific design for the photo-diode in which the active layer is embedded in a p–n junction formed by the upper and lower conductive layers. Preliminary studies have revealed the giant sensitivity of the resistivity of the designed structure to optical illumination at a resonant frequency. We conclude that the fabricated metasurface is highly promising for device applications in optoelectronics.

## Data Availability

Data are contained within the article.

## Appendix A

An SEM image of a typical thin porous TiO_2_ film fabricated via the laser ablation method is shown in Figure A1a. To encapsulate a photosensitive conductive network into a TiO_2_ matrix with gold NPs and sp-carbon nanowires, we deposited a thin layer of sp-carbon dopped with gold nanoparticles on the top of TiO_2_. Gold nanoparticles are clearly visible as black areas in Figure A1b. They were prepared via the laser ablation method in distilled water [16]. Metal NPs formed into liquids via this method are typically encircled by double-containment layers [27]. Thanks to the presence of this layer, gold nanoparticles may stay electrically charged for a long time. We used shungite as a precursor for the synthesis of sp-carbon threads because it is usually free from impurities. The ratios of the concentrations of carbon to gold and gold to water were taken as C:Au:H_2_O = 10^−2^:10^−4^:1.
